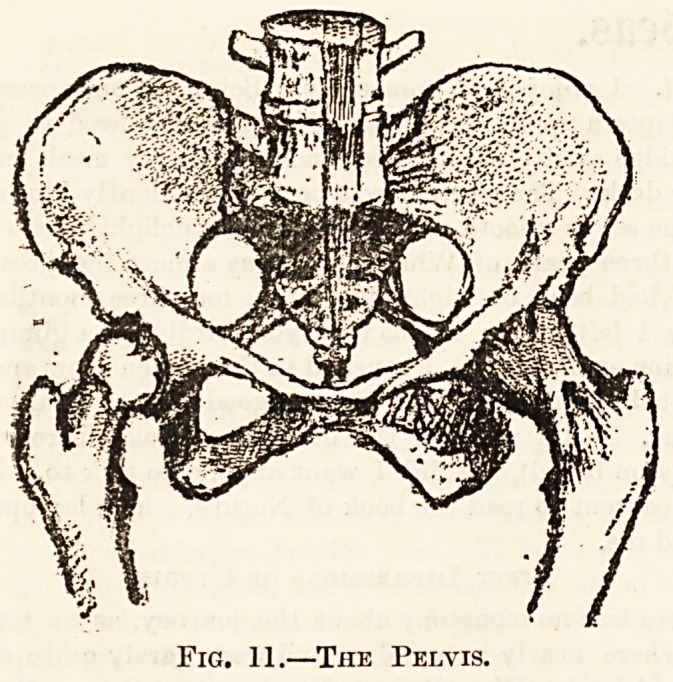# "The Hospital" Nursing Mirror

**Published:** 1900-05-12

**Authors:** 


### "The Hospital" Nursing Mirror.

Being the Nursing Section of "The Hospital."

[Contributions for this Section of "The Hospital" should be addressed to the Editor, The Hospital, 28 & 29, Southampton Street, Strand, London, W.C., and should have the word "Nursing" plainly written in left-hand top corner of the envelope.]

### Notes on News from the Nursing World.

### THE QUEEN AND THE RED CROSS NURSE IN EMBRYO.

Thanks to the good offices of Mr. Chamberlain, a letter written by a little Canadian girl of eleven years old to the Queen was allowed to reach Her Majesty. The letter contained the following passages: "My schoolmates and I want to tell you that we love you because you are our Queen. We love you, too, because you were so sweet and kind to our Canadian soldiers. If I were a man I would go to South Africa and fight for you. If I were a big girl I would be a Red Cross nurse." There is no reason why the latter aspiration of Miss Frances Burt should not be realised when she is a few years older. Meanwhile, she has doubtless derived great pleasure from the gracious reply of the Queen, who expresses herself "touched to receive from her little Canadian subject such a warm-hearted expression of loyalty and of interest in the brave troops united in her army."

### MISS FLORENCE NIGHTINGALE.

Every one of our readers will, we are sure, heartily join with the Nightingale Nurses—who are sending an address of congratulation to Miss Florence Nightingale—and with ourselves, in wishing the illustrious pioneer of trained nursing many happy returns of her birthday. Miss Nightingale completes her eightieth year on Saturday; and, owing to the fact that she enters upon a new decade during the progress of the war, the occasion is one of special interest. Not only members of the nursing profession, but the whole nation, will endorse the hope that Miss Nightingale may be spared to us for many years to come.

### AMATEURS AND ARMY NURSES.

A correspondent of the *Daily Graphic*, who describes herself as an army nurse in South Africa, makes the following astounding statement: "Nurses from Kimberley, Johannesburg, and Cape Town sit with idle hands, many almost destitute, while amateur nurses take salaried positions. Some of them give their services, others have Government posts and receive Government pay." We are not aware how far it is true that there are capable nurses in South Africa "almost destitute," but the definite assertion by an army nurse that some of the lady amateurs who have been so severely censured by Mr. Treves hold Government posts and receive Government pay, is quite incomprehensible. As a matter of fact, the whole of the war nurses sent out from England have been admitted to the Army Nursing Reserve Service, and their training has therefore been subjected to the most careful investigation. It is easy to make general statements of the kind we have quoted, but unless or until an individual instance of the appointment of an amateur as nurse in a military hospital can be proved, they must be regarded as absolutely illusory.

### NURSING AT MODDER RIVER.

An Army Nursing Reserve Sister, who nursed the late General Wauchope's nephew, and says that now that he is better he is always sending her up things for her patients, writes to us as follows: "We were sent to Modder River to nurse an epidemic of typhoid, and indeed we had our work cut out. The first ten days we lived in a railway carriage, as there was not even a tent could be spared. After that they made us up a mud hut which is still our dwelling-place, and is in its way a real palace. At one time there were 80,000 troops here and typhoid just rampant, so you can imagine what our work was like, nursing bad cases in tents, some of the patients lying on stretchers on the bare sand. The work was really fierce, but I would not have given it up for any post in the homeland or elsewhere. Now things are easier and lighter, and we get many little comforts from the base hospitals. The other day I went up to Kimberley (only 25 miles away) just to be able to say I was the first army sister to enter after the relief. A few days after we came here one of the artillery officers took us up to our outpost lines where we saw the big naval guns fired four times at the enemy only two miles away. We also held one of the shells before it was sent off. We then looked through the big naval telescope and saw the Boers distinctly coming in and out of their trenches. Since the relief of Kimberley we have visited Magersfontein. It was most interesting; the Boer trenches looked much better than ours. I have quite a collection of war trophies. Now we are on ordinary rations, the same as the officers and men, namely, $1\frac{1}{4}$ lb. of meat, 1 lb. of bread, two teaspoonsful of tea, the same of coffee, 2 oz. of sugar, pepper, salt, and 2 lb. of wood a day. The officers constantly give us little dainties which they have sent them from home."

### NURSING ON THE "NUBIA."

The nursing sister in charge of the wounded on board the "Nubia" gives some interesting particulars about her patients. There were 150 cases carried on board out of 323, and under 50 had to be carried off at Southampton. There were four deaths on the voyage, though most of the men greatly improved. The very strange case of Private Kibby, of the King's Royal Rifles, is described by the nurse. Kibby was wounded in the knee at Pieter's Hill on the evening of Thursday, February 22nd. He lay where he was all night, still within the Boer lines. On the Friday morning he began trying to crawl to water. A second bullet went through the calf of the same leg. On Friday evening a third went through his shoulder. On Saturday morning a fourth, from our own men (unintentional—but he was right among the Boers) went through the fleshy part just above the wrist. On Saturday evening a Boer, at 30 yards, deliberately fired into his chest. On Sunday morning he was found and carried back! The sister verified the actual wounds, and adds that "the poor man was without food or drink the whole time." The only complaint the hero himself makes is that, as all the five bullets went clean in and out again, he has not one to keep as a relic.

### NURSES AND THE NAVAL BRIGADE.

"There was abundant evidence," says a correspondent, "that a goodly number of nurses saw the 'Powerful' men in London on Monday. Those who witnessed the march of the sailors on their way to the Horse Guards gave one the impression that they had been on duty all night, and that it was only by the sacrifice of their well-earned rest that they had secured a glimpse of the heroes of Ladysmith. I was much amused at finding a stern policeman in Whitehall, who had three times cleared out a piece of vantage ground, finally allowing it to be exclusively occupied by two nurses in the uniform of their hospital. In the afternoon the nursing contingent was much larger, but I again observed a kindly disposition on the part of the public to do their best to secure them a good view. As one rough man remarked, ''Ere, you chaps, make room for nurse in front. There's lots of her sort taking care of Tommy now.'"

### THREE POINTS OF THE COMPASS.

The difficulties of workhouse infirmary nursing continue to be demonstrated. The Scarborough Board of Guardian, who appear to be perpetually in hot water with their nurses, have advertised for four assistant nurses and four probationers to appear before them on May 24th. This determination was arrived at in consequence of the resignation of three nurses in a week, and as the result of a warm discussion. In the course of the latter Mr. Wellburn attempted to compare the difficulty of the Guardians in obtaining nurses to that of the farmers in obtaining agricultural labourers! He was promptly called to order, but the fact that such a comparison should have been made helps to explain why the best nurses avoid the workhouse infirmaries. At Bideford, in the far west, where the same complaint of the scarcity of nurses is made, the Guardians seem to think that the remedy will be found when the new hospital is built; but perhaps their refusal to offer more than £35 a year for a nurse has something to do with it. In the east, at Norwich, the constant changes in the nursing staff are a cause of trouble to the Guardians, and in this case it is urged that the accommodation for nurses is very inadequate. Here the separation of the infirmary from the workhouse proper and the abolition of restrictions upon every movement similar to those obtaining in barracks, seem to offer a palliative. At the moment of writing there is no fresh difficulty in the south.

### HEROISM OF A NURSE.

Last week a sad disaster occurred in Perthshire. About a year ago Nurse Syme gave up general work in order to take the post of staff nurse in an asylum, and on Tuesday she was walking out with two of her patients, one of whom threw herself into the river Tay outside the banks of Murthly Asylum. Though the river at that point is 20 feet deep, the nurse followed her in the hope of saving her, but the current was too strong, and both perished. No doubt other nurses would do the same under similar circumstances, but the act of heroism deserves mention. As the correspondent who communicates to us the facts of the case says, "she was faithful to the end."

### THE DERBY NURSES.

At the thirty-fifth annual meeting of the Royal Derby and Derbyshire Nursing and Sanitary Association a highly satisfactory report was adopted. The staff now numbers 72 nurses, of whom 47 are engaged in private, and 10 in district, nursing, while 14 probationers are in training. Last year five nurses were married, two accepted hospital appointments, and two left in order to take up private nursing. A feature of the report is that the board were able to divide among the nurses as bonuses and percentage £321. The district nurses and a superintendent paid nearly 30,000 visits during the twelve months, and sat up 58 nights at various cases. Dr. Ogle, in referring to the noble work in which nurses generally are engaged, said he should like to see something done in connection with the Derbyshire Royal Infirmary to commemorate the name of Miss Florence Nightingale. At the close of the meeting the Mayor distributed Bibles to the six nurses who had been on the staff of the association for seven years—the Misses Brimson, Hodgkinson, Cash, Routledge, Wilson, and Wainwright.

### A GUARDIAN AS NURSE.

The Wolverhampton Board of Guardians were astonished the other day at the appearance of one of their lady members—Miss Walker—in the uniform of a nurse. In reply to questions addressed to her by her colleagues, she explained that she had only the moment before left the hospital wards of the workhouse, where she had been ministering to the needs of the sick poor. Miss Walker is not an amateur. Some time ago she obtained a hospital certificate, and, on hearing that a number of Wolverhampton nurses were down with influenza, she at once donned her uniform and set to work in the infirmary. The Guardians, in their gratitude to her for coming to the rescue of the institution at the critical moment, voted her their thanks; but Miss Walker disclaimed all desire for praise, and assured them that her work had been a labour of love.

### THE IMPERIAL YEOMANRY HOSPITAL FUND.

Lady Georgiana Curzon sends us the first report of the Imperial Yeomanry Hospital Fund, which gives an excellent description of the steps taken to establish the hospital, and of the arrangements made for sending out the equipment and staff. Happily, the response made to the original appeal of Lady Georgiana and Lady Chesham, on December 29th last, greatly exceeded their most sanguine hopes, and at the present time the sum total of the fund is upwards of £127,000. Judicious use appears to have been made of the money subscribed, and its results are a bearer company in the firing line, a field hospital, and a base hospital. At the base hospital the staff includes 19 doctors, 10 surgeon dressers, 40 nurses, 10 wardmaids, and 76 St. John Ambulance men; there is a staff of 70 at the field hospital, and the bearer company musters nearly 100 persons. According to Mr. Fripp, the senior surgeon, the work done by the nurses has so far been of the most admirable character.

### SIX MONTHS' TRAINING.

Some very complimentary things were said at the annual meeting by the Countess of Warwick and other speakers about Sister Mary, the Matron of the Home of the Essex Cottage Nursing Association. The association was founded in 1894, "to train and supply cottage nurses for the poor," and the home is at Leytonstone. We do not wish, in any way, to depreciate any useful work which is done under the auspices of the matron, but we share the view of those who, as Lady Warwick observed, "consider that the length of training is too short for a nurse to attain anything like proficiency." A woman cannot possibly be trained to be a competent nurse in six months, and a broad distinction should always be maintained between "cottage" and "trained" nurses.

### JUBILEE HOME FOR NURSES AT NORTHAMPTON.

Last week the Countess Spencer laid the memorial stone of the Jubilee Home for Nurses at Northampton, which is being erected by the local fund raised in 1897 for a memorial of the Diamond Jubilee. In addressing a large gathering after the ceremony, Lord Spencer said that nothing would be more agreeable to the Queen than the erection of a nursing home as a memorial of her 60 years' reign. "Many changes," he continued, "had taken place during that period, but none was greater or more beneficial than the manifest improvement in the arrangements for tending the sick and wounded and suffering." The speech concluded with a tribute to the excellence of the work done by the nursing institution.

### NONCONFORMISTS AND THE BRISTOL DISTRICT NURSING SOCIETY.

The Rev. Thomas Lloyd, Congregational Minister, Colwyn Bay, writes us as follows: "I have just seen in your admirable and most interesting issue of the 14th inst. a sentence which, I believe—quite unintentionally on your part—makes an unfair reflection upon Nonconformists. Alluding to the Bristol District Nurses' annual meeting, you say, 'It is a matter of surprise that some Nonconformist churches refrain from giving any support to the District Nurses' Fund,' &c. This notice would lead your readers to conclude that the 'Nonconformist' churches referred to are the only offenders in this matter, but are they? Are there not 'some' of the Anglican churches that refrain from giving this support?" In regard to this we may say that the only reason why we mentioned the Nonconformist churches was that, according to the report in a local paper, complaint was made at the annual meeting of the Bristol District Society that some of them refrained from giving it their support. Nothing was said about the Anglicans. If the representatives of any of the Anglican churches in Bristol have declined to support the organisation, though their members participate in its benefits, our remarks would, of course, apply equally to them.

### "NURSE SUNDAY" AT TORQUAY.

A new departure has been made in the interests of the Torquay Nurses' Institution in the shape of a "Nurse Sunday" collection. The co-operation of the clergy of all denominations was happily secured, and, as comments have been made in these columns respecting the failure on the part of Nonconformist ministers to support the Bristol Nursing Association, we are glad to notice that, in the case of Torquay, Dissenters have been to the fore. The Rev. W. Emery, of Upton Vale Chapel, devoted a sermon to the work of the Nurses' Institution, and mentioned that over 18,400 visits had been paid at Torquay to 357 cases during the past year. "Who," he inquired, "could estimate the value of 18,400 acts of service that brought healing and brightness in the place of disease and sadness?"

### HOWARD DE WALDEN NURSES' HOME AND CLUB.

The new Howard de Walden Nurses' Home and Club, 35, Langham Street, will be open for the inspection of visitors on Friday, May 18th, from three to half-past six, and for the use of the Co-operation nurses on and after the Monday following. Admission on the 18th will be by tickets only, which can be obtained before the 17th inst. by sending a stamped addressed envelope to the Secretary of the Nurses' Co-operation. No personal applications for tickets will receive attention.

### MORE AMATEUR INTERFERENCE.

The following remarkable intimation appears in the *Times* of Monday:—

"Miss S. Buchanan-Riddell is collecting funds to send out workers and stores to assist the overstrained nurses in the Natal military hospitals. Her sister, Miss M. Buchanan-Riddell (Sister May) and a few other nurses are now at the hospital outside Ladysmith, where nurses are greatly needed, and the stores have not arrived. It is hoped that sufficient money will be collected to allow of two nurses being sent off in the "Tantallon Castle" at the end of the week. All postal orders and cheques should be crossed, and made payable to Miss Buchanan-Riddell, 9, Sloane Gardens, S.W. In a letter requesting insertion of this appeal Miss Riddell says: 'I am trying to do what I can to help our troops, especially in Natal. My two brothers, Colonel and Major Buchanan-Riddell, late K.R.R.C., have laid down their lives for their Queen, and my sister and sister-in-law, Mrs. H. Buchanan-Riddell, are out there nursing and tending the sick and wounded. They are welcomed by the military authorities, and have for the last five months been working at Maritzburg, and are now relieving the poor starved nurses at Ladysmith, and the camp hospital seems singularly deficient in necessaries since it was relieved.'"

### SHORT ITEMS.

The Princess of Wales has consented to open the National Bazaar at the Empress Rooms, Royal Palace Hotel, Kensington, on the 24th inst.—The annual meeting of the British Home and Hospital for Incurables will take place at the institution, Crown Lane, Streatham, on Thursday, 24th inst., at half-past three p.m., Earl Amherst in the chair.—We are asked by the secretary of the Whitworth Hospital, Darley Dale, to signify that the election of matron to the hospital will not take place for another fortnight.—On Tuesday the Belfast Board of Guardians will appoint a superintendent of the Fever Hospital. The Board, for reasons of their own, decided to advertise only in the local newspapers, so we may presume that their object is not to secure the best possible list of candidates.—At the annual meeting of the Edinburgh and East of Scotland Co-operation for Trained Nurses, this week, Mr. Menzies, the chairman, stated that the committee had decided to adopt the system of co-operation with the view of giving the nurses a larger interest in the work carried on, and that in future the nurses would be entitled to the fees earned by them subject to deduction of a small percentage for upkeed of the Home.—The editors of *Nursing Notes* are organising an interesting little exhibit of "Nurses' Inventions" in the Charing Cross Hospital section at the Woman's Exhibition. This section is not by any means yet in a completed condition, and the appearance of the "inventions" has therefore been unavoidably delayed for a few days.—By the kindness of Lady Randolph Churchill, Bovril (Limited) are sending quantities of Bovril for hospitals and nursing institutions at the front. The hospital ship "Maine," which has started on its second trip to the Cape, carries the first of these supplies.

### Lectures on Nursing for Probationers.

By E. MacDowel Cosgrave, M.D., &c., Lecturer to the Dublin Metropolitan Technical School for Nurses.

### V.—THE BONY AXIS.

The skull is balanced on the top of the spinal column. It is composed of two parts—brain-case and face. The former is very much the same shape in life as in the skull, as the brain-case is merely covered over by a layer of thin muscle and skin. The face, however, is very different, as the large hollow orbits seen in the skull are filled up by the eyes, the muscles that move them, &c. ; and the temporal fossa, behind each eye and above the ear, is filled up by the powerful muscle moving the lower jaw. The brain-case is composed of the frontal bone (at the forehead), two parietal (forming the top and sides), the occipital (forming the back and base), and the two temporal bones (about the region of the ears). The occipital bone has a large opening through which the spinal cord issues from the skull, and two surfaces which form joints with corresponding surfaces on the upper side of the first vertebra. The bones of the skull are joined by sutures—that is, immovable articulations; they are extremely firm, and no amount of pressure from outside will separate them. In a baby's head the bones have not joined, and spaces closed in by membrane can be felt.

In the face are fourteen bones. The most important are the malar, which form the prominence of the cheeks; the superior maxillæ, which form the upper jaw; and the inferior maxilla, which forms the lower. The maxillæ bones contain the teeth. In the first or milk dentition, which commences when a child is about a year old, are twenty teeth; in the second or permanent dentition, commencing when about seven years old, are thirty-two, the four farthest back of these—the wisdom teeth—generally do not appear until nearly twenty years old.

The bones of the brain-case are flat bones, composed of an

Fig 9.—Side View of the Skull.

outer and an inner layer of hard ivory-like bone with an interval of spongy bone between; if a blow be struck with a blunt weapon the outer table may be broken and crushed down on the spongy layer, but if a blow be struck with a sharp point it may break the inner table as well, and drive the rough-edged fragments down on the brain. In such cases trephining—making an opening in the skull to relieve pressure or irritation—may be required; through this opening room is obtained to reach and raise the pressed-in fragments. The lower jaw is the only movable bone of the skull; it is jointed to the temporal bones, and is liable to dislocation. From its exposed position it is also liable to fracture.

The back-bone, or spine, consists of twenty-four separate bones or vertebræ, which are supported on the sacrum and give support to the skull. Its curvature is concave forwards at the level of the chest and backwards lower down. Each vertebra has a solid part, the body, in front; the bodies are separated by discs of fibro-cartilage, which, being elastic, prevent jar and admit of the spine bending without the bodies separating. Behind is a ring through which the spinal cord runs, and a dorsal and two lateral processes project out at the back and sides. The rings are not as deep as the bodies, so there is room for nerves to pass out. The vertebræ are larger and stronger lower down, as they have more weight to bear. They are divided into seven cervical, twelve dorsal, and five lumbar. The cervical, in the region of the neck, are freely movable; the dorsal give support to the ribs, and are fixed by the dorsal processes, which are bent down and lie on each other; in the lumbar region the processes stand out and once more admit of movement. The first cervical vertebra, or "atlas," supports the skull, and is jointed to it so as to admit of nodding; between the atlas and the second, or "axis," is a pivot joint which allows the head to be moved from side to side. The joints between the other bones of the spine are strongly protected by the different processes, so that it is impossible to injure the spinal cord as long as the bones are intact.

Fig. 10.—Vertebral Column—Lateral View.
1-7, Bodies of cervical vertebræ; 8-19, bodies of dorsal vertebræ; 20-24, bodies of lumbar vertebræ; AA, spinous processes; BB, articular surfaces of transverse processes for the tuberosities of the ribs; C, articular surface of sacrum.

The pelvis, or basin, is composed of a right and left innominate or nameless bone, because it resembles nothing in shape; at the back is the sacrum, a heavy triangular bone on which the spine rests, and which is really composed of five vertebræ fused into a single mass; at the lower end of the sacrum is the coccyx, composed of four rudimentary vertebræ. Each innominate bone is made up of three parts, the ilium or flank bone, which can be felt above the hip; the ischium or haunch bone, on which the body is supported when sitting; and the pubes in front. The three bones meet at the hip-joint, each forming part of the cup-shaped depression into which the head of the thigh bone fits. The bones of the pelvis are firmly joined together without any movable

FIG. 11.—THE PELVIS.

joint; when standing, the pelvis is tilted nearly on its lower edge so as to bring the weight of the body in a vertical line through the spine and pelvis to the head of the thigh-bone, and so down to the summit of the arches of the feet.

### On Asepsis.

THE germs were multiplying fast,
When through the hospital there passed
A youth, who met with jest and jeer
Because he made his motto clear,
"Aseptic."

His brow was sad; his eye beneath
Flashed like a falchion from its sheath,
And like a silver clarion rung
The accents of that foreign tongue,
"Aseptic!"

"Beware!" the honorary said;
"They'll heap abuses on your head,
Our micrococci all are dead;"
He murmured with a look of dread,
"Aseptic?"

The nurses love him not; they say
They have to toil both night and day,
They boil and scrub with all their might
In frantic efforts to be quite
Aseptic.

The enraged dispenser gnashed his teeth
And said bad words below his breath:
"The doctor needs perchloride rain
To fill these bottles up again,
A—ss—eptic!"

"Oh, run!" the sisters cried, "and play!"
"You badly need a holiday.
Before you came with marked success
Our burns and wounds we used to dress;
You found them all, you must confess,
Aseptic."

As to the wards at break of day
The nurses filed in full array,
They heard from out the theatre door,
A dying voice ring through the air,
"Aseptic!"

There, in the irrigator drowned,
The hapless house-surgeon they found;
Lifeless but beautiful he lay,
His boots turned upward to the day,
Aseptic. —*A. H. M.*

### First Aid to Gun-shot Wounds.

EXAMINATION QUESTIONS FOR NURSES—RESULT OF APRIL COMPETITION.

THE question was as follows: "Describe how you would deal with a patient brought into hospital on a stretcher, and reported to have been shot in the side?" "Nurse Ruth" takes the first prize; her answer is short and comprehensive, and in the event of a nurse seeing a case of gun-shot wound before it has been examined by a surgeon, her proceedings would be quite correct.

NURSE RUTH'S ANSWER.

"Lift patient carefully in a horizontal position from the stretcher to the bed, having first prepared the bed by putting in a mackintosh and draw-sheet. Undress patient quickly and quietly, cutting the seams of any garments that are tight. Place several hot-water bottles in the bed, and a blanket (warm) next the patient. Keep the head low, and give no nourishment till ordered, as faintness will help to counteract the hæmorrhage by reducing the heart's action. Wash the wound thoroughly with a warm antiseptic lotion, removing all clots of blood. If the bleeding is very profuse, pack the wound with antiseptic gauze, placing a firm pad of lint and wool over the wound, and hold it in position until the house-surgeon comes."

HONOURABLE MENTION AWARDED TO THREE.

Very few answers have been sent in this month, and no second prize will be awarded; but Nurse Hancox, Nurse Mary, and Nurse Scott have secured honourable mention. These answers are all good, but they go a little too far in dealing with a patient who has not yet been seen by a superior authority. I hope that more answers will be forthcoming for next month.

QUESTION FOR MAY.

What would you do in a case of severe burn where the doctor lives several miles away, and probably may not arrive for several hours?

RULES.

The competition is open to all. Answers must not exceed 500 words, and be written on one side of the paper only. The pseudonym, as well as the proper name and address. must be written on the *same* paper and *not on a separate sheet*, Papers may be sent in for fifteen days only from the day of the publication of the question. Failure to comply with these rules will disqualify the candidate for competition. Prizes will be awarded for the two best answers. Papers to be sent to "The Editor," with "Examination" written on the left-hand corner of the envelope.

N.B.—The decision of the examiners is final, and no correspondence on the subject can be entertained.

In addition to two prizes, honourable mention cards will be awarded to those who have sent in exceptionally good papers.

### Robbing a Nurse in the East-End.

AT the Thames Police-court James Dooley, aged 22, was charged with feloniously assaulting and robbing Miss Laura Graham, a nurse at the St. George's-in-the-East Infirmary. Shortly after five o'clock on Sunday afternoon, when the prosecutrix was in Ratcliff Street, the prisoner came up and struck her in the chest. She put up her hands to defend herself, and the accused knocked her down and tried to get her purse, which she was holding in one hand. The prosecutrix struggled and got up, but Dooley again knocked her down, and, having got possession of the purse, ran off with it. Information was given to the police, and the same night the prisoner was arrested. Dooley was placed among nine other men, and Miss Graham at once picked him out as the man who had assaulted and robbed her. A lad named Robert Sullivan said he knew the prisoner by sight, and on Sunday afternoon saw him running away, followed by the prosecutrix, who called out "Stop thief!" Mr. Mead, having heard that the prisoner had been previously convicted, committed him to the Central Criminal Court for trial.

### Across the Seas.

### NURSING IN CYPRUS.

By a Correspondent.

"Ask Nurse Crossman, she will be able to tell us; she knows everything."

This very flattering remark fell on my ears one morning as I was placidly finishing my dinner in the nurses' dining-hall of one of our biggest London hospitals.

The clatter of knives and forks and hubbub of voices had ceased for a moment as the home sister rapped on the table to ensure silence before making an announcement of some sort. It broke out louder than ever as soon as the sister stopped, and hearing my name mentioned I inquired what it was about.

"Cyprus, Nurse; where is it?" exclaimed several voices together. "A nurse is wanted there for the Government hospital. It is sure to be in some remote corner of the globe," they added. "Colonial appointments generally are."

I assured them that it was no remoter than the Mediterranean; but at the same time I could not help thinking that, in spite of the visions of blue sky and sunshine conjured up by the Mediterranean, the name had a curious ancient flavour about it, reminding one of St. Paul and the "Acts of the Apostles."

It was the middle of November, and we had been enveloped in thick fogs for a week, so I determined to try for the chance of nursing in summer climes. I put my name down as an applicant for the post, and was lucky enough to get it.

### Ophthalmia in Cyprus.

I was given about three weeks to get my things ready, but though Cyprus is barely a fortnight's journey from England, no one seemed to know anything about it, so I procured my outfit in blissful ignorance of what was really required. A knowledge of ophthalmic work was necessary, so I filled up the intervening time in the ophthalmic wards, brushing up my eye work. A first-class passage was taken for me by the Crown Agents in a Messageries boat, which sailed from Marseilles, and I was also provided with a first-class railway and boat ticket from London. It was a most uncomfortable journey, bitterly cold, and snowing all the way from London to Paris. I was dead tired when we arrived at last at Marseilles, and was only too glad to put myself and my luggage into the hands of one of Cook's men, who directed me to a good hotel, where I had a wash and brush up and an excellent breakfast, after which I sallied out for a walk, Cook's man having promised to call in the afternoon and pilot me safely on board. I had never been on the continent before, and could scarcely speak a word of French, so Cook's man was a sort of harbour of refuge or guardian angel to me. He turned up in the afternoon and saw me and my luggage safe on board the "Niger," and I am afraid I gave him a much larger tip than I ought to have done, but he didn't ask for anything, and I was so grateful at the time.

We were a very mixed crowd at dinner that evening. French, Greeks, Armenians, Jews, Turks, Sisters of Mercy, and soldiers, all chattering volubly with that light-hearted gaiety which seems natural to foreigners. I was the only Englishwoman, and felt very lonely at first, but I soon made friends with a Greek lady who sat next me, whose English was about as bad as my French, and we were soon laughing merrily at our desperate efforts to understand each other. The next day we were at the Piræus, the port of Athens. I was very anxious to go ashore, but we did not get in till very late in the evening, and the captain said there would be no time to do it next day, so I had to content myself with watching the town from the deck.

All the saloon passengers except the French Consul and his wife left us here, and they departed at Salonica, so from Salonica to Larnaca I had the whole of that big boat to myself. I enjoyed it immensely. Being the only passenger the Captain spoilt me dreadfully. I was allowed to go on the bridge as often as I liked, and had all my meals on the upper deck. The weather was perfect, brilliantly blue skies, and the sea as smooth as a mirror. The delightfulness of it after three years of Whitechapel was almost inexpressible, and I had been on night duty, too, for three months just before I left, which means that you hardly get a glimpse of sunshine or blue sky. I revelled in the golden glow and the fresh balmy breezes. It seemed so good just to be alive and breathe. I did not feel the need of a book (there was no library on board), nor did I want anyone to talk to. I was quite content to read the book of Nature, which lay open all around me.

### First Impressions of Cyprus.

There was no monotony about the journey, as we touched somewhere nearly every day, and were rarely quite out of sight of land. We called at Rhodes, Scio, Samos, Patmos, and several other islands for cargo, wine chiefly; they were all beautiful, in fact, every prospect pleased, but only man was vile, for, alas! at Patmos I lost a valuable rug, stolen by a degenerate Greek while I was at dinner. It was always chilly in the evenings, and as that was the only rug I possessed, I was obliged to shiver for the rest of the voyage. Fortunately I hadn't to shiver very long; we were in Larnaca Roads in three days.

The first view one gets of Cyprus is not prepossessing—not, at any rate, from that side of the island. Larnaca is about the ugliest spot in a beautiful island, and it was with a feeling of disappointment that I looked at its low, flat shore, with a few straggling houses scattered about and scarcely a tree or a bit of green to be seen anywhere. I had not much time, however, to gaze at the scenery, as a boat was coming alongside; and presently I heard someone inquiring for me. He was a messsenger from the Commissioner of Larnaca, who was waiting on the pier to receive me. On landing he invited me to luncheon, but as I had only just breakfasted and was very anxious to get on to Nicosia, I declined his invitation. We proceeded to the Custom House, where, after signing a declaration that I had no plants, flowers, or fruit with me, I was suffered to depart. My boxes and I were hoisted into a ramshackle old diligence, and away we went rattling on a twenty-six mile drive to Nicosia. The diligence was a roomy, lumbering old vehicle, capable of holding six comfortably inside; the horses, poor, weedy, half-starved things, were harnessed four abreast, the harness being tied together in several places with bits of string. The smell inside was awful, though I had all the windows open, and as we drove mile after mile, through barren, stony tracks of land, without a sign of human habitation or life, I began to wish myself back in old England.

### Arrival at Nicosia.

Presently the country began to look a little more cultivated; green fields took the place of stony wastes, and we passed flocks of sheep and strings of camels sailing silently along, and a few more miles brought the palms and white shining minarets of Nicosia into view. The town is surrounded by a wall, and is entered by four gates guarded by "Zaptiehs" (native policemen), both Turks and Greeks, attired in most picturesque uniforms. They are commanded of course by English officers. We were soon rattling through the Larnaca gate, and pulled up at the chief medical officer's house. I was very weary, and the kind, hearty welcome of the good old doctor and his charming wife cheered me greatly. They insisted on my spending the night with them, and the next morning the doctor drove me down to the hospital, which was about a quarter of a mile outside the town.

### The Hospital.

It is a substantial stone building, standing in rather extensive grounds. The original idea was to build the hospital in three blocks, connected by corridors, the centre block to contain operating-room, out-patients' dressing-room, dispensary, doctors' consulting-room, and board-room, while the two wings were to be the male and female native wards. No provision was made for accommodating European patients, as, until then, Europeans had been nursed in their own homes by two English nurses, sent out by the Cyprus Society. Unfortunately, owing to lack of funds, the left wing has never been built, so the female patients are lodged in the upper storey of the right wing, each ward having nine beds. To the extreme right another single-storey block has been built for eye cases. This has been done by private subscription, raised in the Jubilee year. It has eight small wards with two beds each, and a dark-room for use of the ophthalmoscope. The walls are tinted grey, and altogether it is a perfect little place, the only fault being that as it is isolated from the main building it means a great deal of exposure to the weather for the nurses. In addition, right at the other end of the compound are two small isolation wards with four beds.

### That "Pesky" Mosquito.

By a Nurse in a Malarial District in the "Far West."

One sees and reads so much nowadays of the mischief caused by those wretched little pests, mosquitos, both by causing malaria and being the mediums for the carriage of other diseases, to say nothing of the awful discomfort they cause to the sick, who are at times too feeble to brush them away, that it may be of interest to some of your readers to know how the little "abominations" can be banished from the sick room, and, indeed, from the whole house.

Most colonial and tropical houses have their windows and doors protected by "mosquito netting," either of fine wire or cotton. This, like many other things, often acts both ways, for, though excellent for preventing mosquitos and flies from entering in large quantities, it still has the disadvantage of keeping imprisoned any stray ones who may have managed to sneak through while a door has been opened, when they hide till they can begin their bloodthirsty revels. At twilight they make a last effort to catch a glimpse of our magnificent sunsets, and many can then be exterminated on the windows or curtains surrounding them. It is then requisite to get some old saucers or basins (I say old because of the lasting stain the remedy leaves) and fill with permanganate of potash solution. I place one on each side of my patient's bed. If the room is large, I put one or more in different parts, or should the doors or windows be unprotected by netting—a defect I do not leave long unremedied—I place a larger dish of the solution in the window to greet the unwelcome visitors, and often I have the pleasure of seeing them approach, and then retire in discomfort and disgust.

Next morning I empty and wash the saucers in a basin, which afterwards I carry out and throw into any pools, troughs, and closets that may be near the house. This will not harm chickens, dogs, hogs, nor horses; in fact, is an excellent preventive of cholera in hogs and chickens. In this inexpensive and simple manner I manage to secure comfort and rest for my patient, other inmates, and self. Of course, it needs renewing daily, but by buying it in bulk it is very cheap. For the worth of about a shilling in our money I obtain immunity through a whole typhoid case. This not only keeps mosquitos away, but destroys the egg and chrysalis of the "unfinished" mosquito in their muddy water birthplace. A doctor here tells me that in case of snake or other poisonous bite a hypodermic injection of the solution in the neighbourhood of the affected part, if given quickly, is an admirable remedy and antidote.

### The Modern Treatment of Epileptics.

### A VISIT TO THE MAGHULL HOME.

By a Correspondent.

The other Sunday afternoon I paid a most interesting visit to the Home for Epileptics, near Maghull. On the way I was met by the girl I had gone to see, and a companion in affliction, for the slighter cases are allowed out occasionally in two's and three's. We walked along country roads, past cottages whose gardens were full of fruit trees just ready to burst into bloom, the fresh green peeping everywhere, and a glorious sunshine over all. Presently we arrived at the home, once an old country house, surrounded by fields and trees. Not far off in the same grounds is the men's establishment—a modern building, with a hall attached for Sunday services, &c. The inmates were walking round the grounds of their respective houses or sitting in lounge chairs in the gardens. One poor child sat listlessly in a chair, her face badly cut and bruised. I was told that as she had fits frequently, and always fell on her face, she was never "out of the wars," and consequently got the name of "old soldier." Some of the girls seemed very bright and happy; others quite simple.

### The Home.

My friend showed me round with great pride. We went into the old kitchen garden, surrounded by high walls for fruit trees. In here the patients each had a little garden, and in the greenhouse were boxes with seeds forcing for being transplanted by-and-bye. In one corner was a rustic bower made by a woman that was to have creepers trained up it presently. Through a door in the wall we passed into a green surrounded with trees, where were hens, one proudly clucking to a fluffy little family. The stable and shippon were locked, so I could not see the cows and horses. Near by was the laundry, with five or six wringing machines—just imagine the noise of them all going at once!—and capital drying and airing apparatus. There are no house servants, the work and washing all being done by third-class patients, superintended by nurses, a laundress, and a gardener for the gardens. The cleanliness and beautifully scrubbed woodwork are very creditable. On the basement floor—which is really a ground floor—are three cheerful sitting-rooms, third-class. In one hung a bird and cage, a patient's pet. The next two floors are devoted to first and second-class and nurses' quarters, and the top to thirds' bedrooms. One large room had twenty-five beds (I believe), set head to foot, with respectable distances between. One room of six beds is devoted to Liverpool Union cases, who are sent in well provided with suitable clothes and neat dark-red dresses.

### Bright and Smiling Patients.

Descending the stairs we met a crowd returning from the afternoon service. It was difficult to realise that these bright and smiling women were epileptics. Putting me into a small room my friend disappeared, and presently returned with a welcome tea-tray. As she presided, she remarked, "The matron is away ill, and the head nurse is her deputy, and kept busy between the two houses. We are all very fond of her; she is so just. It is a bad look-out for anyone found imposing on a weaker patient, and she takes such an interest in everyone. The nurses are very good, too. We have dances, the last one fancy dress, devised by ourselves. Lizzie, the girl who came with me to meet you, had a long train to hers, and she could not manage it at all; got hopelessly mixed up when she tried to dance. We are not allowed time to indulge in the "blues" here after the work is over. There are singing lessons three nights weekly, and a night school, which is under Government. I think the aim to keep us well and happily employed is certainly successful." I heartily agreed with her, and when I came away I thought of the difference to the old days when epileptics were shunned and ill-treated as "possessed of the devil," and now have kindly care, so that they are seen at their best.

### Nursing in Las Palmas.

BY A SISTER.

So many inquiries have been made by our readers about nursing in the Canary Islands, that we are sure the following article will be read with interest:—

The winter before last I accompanied a lady patient to the Canary Islands in the capacity of companion-nurse. We went first to Las Palmas, the capital of Grand Canary, and settled down comfortably at the spacious and airy Hôtel Metropole. Of the beautiful sea and mountain scenery I will say nothing, as it does not come within the scope of this little paper.

My patient took a "siesta," after the Spanish custom, during the hottest hours of the day, and I was free then, and generally spent the time exploring the town.

### THE SPANISH HOSPITAL.

Discovering that there was a Spanish hospital in the vicinity, I set out one day to visit it, accompanied by a small party of ladies from the hotel. We got into the noisy, bustling steam tram which passes the hotel door, and were quickly borne along the dusty road till we reached the town. Here we alighted, and, turning up a side street as directed, we walked along past tall white houses with picturesque balconies till we reached one with "Hospital," in stone letters, over the door. This was soon opened to us by a pleasant-looking nun, who, quickly guessing from our gesticulations and broken Spanish that we wished to see over the hospital, motioned us to follow her. We passed through a sort of paved garden, where a fountain played, and where bougainvilleas, hibiscus, and other gorgeous tropical shrubs bloomed luxuriously and cast a pleasant shade.

### THE WARDS.

Our guide took us first to the men's ward, which was large and airy. The patients were all in bed. Knowing so little of their language we could not find out much about the cases; but here and there a bandaged head or arm spoke for themselves. The patients looked pleased to see us, and when the nun told them that I was "enfermera Inglesa," *i.e.*, "an English nurse," they smiled and nodded comprehendingly to me, and admired the long veil from my bonnet. We then went to the women's wards, of which there were two, containing five or six beds each. The beds here were covered with white quilts, those in the other ward being coloured. Some of the women were up and sitting at the windows. The floors were well scrubbed and white, and everything was scrupulously neat and clean. The nuns do all the nursing. Our guide then showed us into a room where we saw a number of tiny swinging cots, all empty now; this was evidently used as a babies' ward when required.

### THE INFANT SCHOOL.

We were then taken up and down innumerable stairs and along a corridor to the infant school adjoining the hospital, where a sweet-faced nun was teaching a lot of dear little dark-eyed children, who stood up and curtseyed gracefully when we entered. We saw the needlework school under the direction of two nuns; here the elder girls were making most exquisite white and gold embroidery. "Para la iglesia," *i.e.*, "for the church," they smilingly told us. We purchased some beautiful and very cheap lace handkerchiefs made by the pupils, and then took our leave with many thanks to our pleasant guide, who seemed gratified at our appreciation of the good work they were doing in this far land. We passed out through the cool garden into the hot street, and finding that we had missed the steam tram we walked back to the hotel along the seashore.

### THE ENGLISH COTTAGE HOSPITAL.

Another day we took the steam tram in the opposite direction to see the English Cottage Hospital, situate in the Puerta *i.e.*, Porte. The nursing staff consists of a matron and one nurse, who were both in and kindly showed us over. There was one bright, airy ward for general cases, and a few smaller rooms containing one or two beds for private or special cases. The patients were mostly sailors who had met with accidents on board ship, or been smitten down by the terrible West African fever, Las Palmas being a coaling station on the way to and from that deadly coast. One man, who was sitting up in a Madeira chair, told us how he had caught the fever down the coast, and having been brought in a ship to the port here, had been carried more dead than alive into the hospital. He spoke gratefully of the good nursing and care he had received, and said he felt he would soon be able to start for England, which he had never expected to see again. Another sailor, a young fellow, was lying in bed with a badly-burnt foot; he was reading an old copy of "The Strand Magazine," and looked quite happy. The English doctors resident in the island attend this hospital. Having seen the pretty rooms of the matron and nurse, we peeped into the cosy sitting-room, used as a committee-room when necessary, and then having been shown the kitchen, where preparations for dinner were in an advanced stage, we took our leave, well satisfied with our visit.

### Presentations.

ALTRINCHAM HOSPITAL.—Miss E. A. Twynam, who for the last eleven years has held the position of matron of the Altrincham Hospital, has been the recipient of a handsome dressing case containing a full set of silver-mounted brushes, &c., with engraved monogram. They were enclosed in a Russia leather box, with a silver plate, inscribed: "Presented to Miss E. A. Twynam, matron, as a mark of respect and esteem by the nursing staff of the Altrincham Hospital, 1900." In making the presentation, Sister Symons, the night superintendent, said she had been asked by the staff to hand over the gift. The pleasure with which she did so was mingled with regret that Miss Twynam was about to leave them. She wished very heartily to testify to the good feeling that had prevailed during the time they had spent together at the hospital. There had been the greatest possible harmony, and the good wishes of the nurses would accompany Miss Twynam on her departure from the hospital. Miss Twynam, in accepting the gift, heartily thanked the staff for their mark of kindness. Dr. Luckman afterwards distributed the prizes and certificates awarded as the result of an examination on a course of lectures on anatomy, physiology, medicine, and surgery, given during the winter session, and in doing so congratulated the nurses on the steady progress they had made, and said it was satisfactory to find that they had evidently done their utmost to acquire the knowledge requisite to fit them for the career they had chosen. The recipients were: Nurses M. H. Morris, A. M. Fulham, E. M. Rees, A. Prouting, L. E. Nolde, A. H. Rollinson, A. E. Roberts.

KIRKHAM WORKHOUSE INFIRMARY.—Miss C. G. Carr, who is resigning her position in the Kirkham Infirmary in order to nurse her mother in a dangerous illness, has been presented with a handsome marble timepiece. During her five years' services she not only won the admiration of the various officials and Guardians, but also of the inmates under her charge, and great was the sorrow of all, especially the latter, when she felt it her duty to resign. The inscription on the clock is as follows: "Presented to Miss C. G. Carr by the Guardians and officers of the Fylde Union, Lancashire, as a mark of esteem, and in appreciation of the faithful manner in which she fulfilled the duties of charge nurse at the infirmary for five years.—Kirkham, April, 1900." Miss Carr received her training at Birmingham.

### The Nurse in Fiction.

Although "The Disenchantment of Nurse Dorothy" (Skeffington and Son) is brightly written, Miss Florence Baxendale's "Story of Hospital Life" is open to the objection that it may convey the erroneous impression that a hospital is an institution which affords particular opportunities for love-making. Miss Isabel Herbert, the matron of St. Bernard's, in gloating over the departure of a probationer whom she disliked, is made to observe to the house-surgeon, "It is really remarkable how girls of all classes flock to hospitals; I suppose they think there is something novel in the life. They nearly all come with such absurd ideas; it is delicious taking them down." Miss Herbert is not a pleasant kind of person, though the bitterness with which the author writes of her, describing her office as the "torture chamber," and her gratification in doing "the devil's work," suggests that if she is drawn from life, the delineation is coloured by a feeling stronger than prejudice. But the account of the heroine's relations with Mr. Hurst and of "Bobbie" Graeme's happy engagement to Mr. Fraser can scarcely fail to encourage the belief which still prevails in certain quarters that the prosaic duties of a probationer in a hospital may be varied by tender passages with members of the medical staff. The fact that Nurse Dorothy is never really happy as the fiancée of Mr. Hurst, and that the passages in their case are scarcely tender, will hardly suffice to serve as a warning to romantic young ladies not to look for husbands in the wards.

Miss Baxendale, however, has evidently a considerable personal acquaintance with hospital life, and some of her sketches of character are admirably done. We greatly prefer Sister Laura not only to the terrible matron of St. Bernard's, but also to the genial lady who presides over the nursing department at St. Helen's. Here is a word picture of the former: "Miss Herbert looked an imposing spectacle as she came sailing along in full nurses' uniform. Her gossamer veil fell below her waist, her cloak floated peacefully in the breeze; she felt, as she noted the admiring and reverential looks of the passers-by, heroic and sublime—a second Miss Nightingale, or at the very least a second Sister Dora"; and there are others still less complimentary. Miss Marshall, the matron of the Children's Hospital, is a very different kind of individual. There was no torture chamber at St. Helen's, and Miss Marshall seems to have had such a dread of being regarded as an ogre that she did not care to be addressed as "matron." This is going to the other extreme. Moreover, apart from the question of discipline, which may or may not be slackened by the use of the name instead of the official designation, some of the most kindly and gracious of women have been known as "matron." It is a title to be proud of, not to be ashamed of.

Sister Laura is depicted as a thin woman of uncertain age. "Sometimes she looked barely thirty, at other times, when worried or not very well, she looked over forty. She had a good deal of fair hair streaked with white, insignificant features and light eyes; not a pretty woman at all save for her smile, which was beautiful, and was apt to break out like sunshine on a wintry sky." The heroine had heard her criticised rather more freely than kindly by the nurses, but her own experience soon satisfied her that they were wrong. Upon the arrival of Dorothy she gave her new assistant a keen look, said "Good morning," and then turned away and began to busy herself over certain matters that the sisters usually left to their subordinates. "Dorothy found that her morning's work was made considerably lighter by this help. As the day passed she was sensible of a feeling of peace; she received no peremptory orders, nor did she hear any jarring words. The sister was patient, and expressed her wishes clearly and in a quiet manner. The very patients, poor souls, seemed more cheerful and serene than in any other ward she had worked in. They took their tone from their head nurse, and addressed the nurses civilly, as she did, and any person who has worked within hospital walls knows what a difference that makes, the difference, indeed, between happiness and unhappiness." As time went on Dorothy became greatly attached to Sister Laura, and the attractive personality of the latter reveals itself at many stages in the story. Here is a description of her which again suggests that her counterpart in real life is known to the author; "She was a splendid nurse and a good woman. She would have managed excellently a large hospital, but she had always been quiet and unassuming. She had no influential friends, nor was she handsome, and so she had been kept in the background."

Nurse Dorothy herself is interesting, and her excellent qualities render it all the more regrettable that she allowed herself to come under the influence of such an exceedingly objectionable personage as the surgeon Hurst, who certainly is not a typical member of the medical profession. She pays dearly for her mistake, and those who follow her history will be glad to find that she eventually escapes from a position which was always embarrassing and often painful. After she left St. Bernard's Hospital she went to a nurses' home and engaged in private work with varied experiences. In one instance the daughter of the house disliked her, and saw "cheek" in her most innocent words and actions; but in the end she reached the haven of contentment, a result which, it is hardly necessary to add, seldom occurs through the medium of their professional duties to nurses quite as sweet and charming as Nurse Dorothy.

### The Nurses' Bookshelf.

[We invite Correspondence, Criticism, Enquiries, and Notes on Books likely to interest Women and Nurses. Address, Editor, The Hospital (Nurses' Book World), 28 & 29, Southampton Street, Strand, London, W.C.]

Gynæcological Nursing. By G. A. Hawkins - Ambler, F.R.C.S. (Scientific Press, Ltd., 28 and 29, Southampton Street, Strand, W.C. Pp. 96. Price 1s.)

There is so little comparatively written for nurses on this important branch of their profession that we are inclined to think that Mr. Hawkins-Ambler has conferred a service on them by the publication of this useful little work. Very explicit are the directions he lays down for the guidance of those in charge of gynæcological cases, and very rigidly he insists on the importance of faithfully carrying out all antiseptic precautions. The cultivation of the "antiseptic conscience," as he aptly describes it, demands no little care and self-denial on the part of a nurse. We say "self-denial" advisedly, as it means perpetual watchfulness and conscientious performance of the details of the most trifling offices. Proceeding from the hypothesis that "a good gynæcological nurse must first be a good general nurse," the writer goes on to enumerate the various qualifications that are indispensable to insure success. The chapters on douching and examination are among the most useful in the book and alone should command the attention of all trained nurses, who always at some period or other of their career are called on to undertake such offices. There are also some valuable hints on the application of tampons and plugs, and on the use and care of catheters. That on the after care of cases is among the most valuable of the concluding chapters, and there is a useful formulary appended which cannot fail to be of real service to the reader. It is with confidence that we recommend this little book to all workers in this special branch of nursing. The price is modest enough to bring it within the reach of all.

### Echoes from the Outside World.

### AN OPEN LETTER TO A HOSPITAL NURSE.

FOR the past few seasons it has been said, year after year, that the Academy has been the worst known. It is quite refreshing, even if all are not agreed, to hear from most a different tune, and one is doubly pleased to find that the general opinion leans towards praise this year, because so many of the artists, during the winter, so generously gave of their time and their talent to the war funds. Assuredly there is much to interest at the exhibition in Piccadilly, and though I can only touch on the fringe of such a huge subject this week, I must mention the pictures likely to be talked about. But before I go any further, let me advise, when you go to Burlington House, that you take with you the particulars of the competition which has been arranged by the *Daily News*. It is simply to give an expression of opinion as to the best pictures of the year, and full particulars appear in the paper. If you don't win the £50 prize, you may get a £5 note.

FIRST amongst the celebrated canvases comes Mr. Orchardson's "Windsor Castle, 1899," in which he treats portraiture in a most charming manner. The Queen is seated in her arm chair with a small work table on one side. Towards her comes little Prince Edward of York, offering a large bouquet of flowers. He looks a brave, bonnie wee man, and no wonder that his father and his grandfather, who are looking on at the picturesque incident, appear happy and proud. The picture has been painted for the Royal Agricultural Society. Another interesting group is "Lady Elcho, Mrs. Adeane, and Mrs. Tennant," the three daughters of Mrs. Percy Wyndham, and sisters of Mr. George Wyndham. All three ladies, who are great beauties, are dressed in glittering, sheeny white, with here and there a glimpse of softening gauze. The cushions of the white sofa are green, so are the walls of the drawing-room, in which are great magnolias, and the subtle combinations of harmonious greens and whites, with shadowy greys and soft flesh tints is one of the cleverest things which Mr. Sargent has ever done Mr. Frank Dicksee contributes a picture which is sure to be widely discussed, entitled "The Two Crowns." A king, attired in golden armour, rides upon a richly-caparisoned white charger down a mediæval street. Before him the maidens strew roses, behind him walk armed men, and his triumph seems complete. Suddenly his eye catches sight of another King, who, crowned with thorns, is hanging from a cross, a presentment of the well-known crucifix figures which are so often seen by Continental roadsides. The contrast is obvious, and the suggestion must appeal to all, but I wish that the face of the earthly monarch had been more expressive of the thoughts which must be traversing his mind.

ON Sunday the German Crown Prince came of age, having attained his eighteenth birthday. He is half a head taller than his father, is said to have very charming and simple manners, and knows an immense deal about modern warfare. He has been a lieutenant since he was ten, has had a miniature fortress, correct in every detail, built in his garden, so that he might learn whilst he played; and now his time has come, as the Kaiser says, to "go on duty." I am sure we all wish him a long and prosperous career, but I am so glad I was not born Royal.

THE present craze for wearing little medallions containing the portraits of celebrated persons has its disadvantages as far as the celebrities themselves are concerned. We daily gazed upon the features of Sir George White for so many months whilst he was shut up in Ladysmith, that now, though free from the attentions of the Boers, he is by no means released from the attentions of the British. Even the schoolboys in the street recognise the rugged, kindly features as soon as they appear, and practically Sir George is safe nowhere. When he visited the Hippodrome the manager had to come to his assistance to prevent him being too closely besieged; at the private view of the Academy he was stared at far more than Mr. Orchardson's "Windsor Castle," or Mr. Dicksee's "Two Crowns," and found it difficult to study a single canvas as closely as he was studied; whilst at the Steinway Hall, where he presided over an international exhibition of sword play one evening, he had good-humouredly to protest at being made too much of. He was cheered vociferously, the crowd declared in song that "he is a jolly good fellow," and then, notwithstanding that he came on conditions that there should be no speechifying, he had to address the audience. I was amused to find him using that very American word, the verb to "enthuse," and declaring that the reception accorded him had so "enthused" him that he felt he might say a few words. Those words, too, were quite to the point, for Sir George is almost as good a speaker as a soldier.

NEXT to the King of Sweden and Norway, the most illustrious of our foreign visitors is Prince Kotohito of Japan. His Imperial Highness, who was one of the guests of the Academy on Saturday evening, is hardly likely to leave our shores without visiting the little institute at Tilbury, presided over by Miss McLean, where Japanese sailors are always made welcome on their arrival from the Land of the Rising Sun. Miss McLean has spent many years in Yokohama, and thoroughly understands the Japanese language and character. She has a two-fold object in view in interesting herself in the blue-jackets of the Imperial Navy and the men of the mercantile marine. One is educational and to afford pleasure, and the other is to keep the strangers from Japan out of our public houses. Miss McLean, who personally conducts the sailors through the streets of London, says that of all the sights they appreciate the Zoo stands first. "They have nothing like it at home, and they can never see enough of it." Other of their favourites are the fireworks at the Crystal Palace, the arms and armour at the Tower of London, and the model of the Battle of Waterloo at the museum of the United Service Institution. Miss McLean finds them singularly intelligent, and they know more about great Englishmen like Nelson, Wellington, and Gordon than many English people. The dome at St. Paul's, the monuments at Westminster Abbey, the mummies at the British Museum, especially excite their wonder and admiration.

ALTHOUGH the good old adage says "Ne'er cast a clout till May is out," as soon as the sunshine comes in real earnest his persuasive powers are so great that old saws and sayings are forgotten, and one is obliged to turn one's mind to questions of new garments. Stitching and strapping are a great feature of all the spring productions. The cloth jackets are either stitched with white or self colour, or they are strapped with cloth or silk; and tailor-made dresses are treated in the same way. A very effective dress which I saw the other day, and which could be copied quite inexpensively, was of black fine cloth trimmed with graduated straps of black and white cloth, collar and revers being of white cloth with the narrow straps of the black and white laid rather closely together. The vest was of soft white silk. I surreptitiously stared hard at the wearer of the dress, because it struck me how stylish she looked, and yet what a serviceable foundation there was upon which to erect a more useful dress as soon as the white had got soiled. It would be so easy to retrim such a garment to advantage.

### The Male Nurses of the "Maine."

WHAT LADY RANDOLPH CHURCHILL SAYS.

Certain complaints having been made in the columns of a weekly contemporary as to the treatment which several of the male nurses had received on board the "Maine," a representative of the "Nursing Mirror" says: "I called upon Lady Randolph Churchill at her residence in Great Cumberland Place on Monday to ask her if she cared to make any statement on the subject. I began by inquiring whether Lady Randolph had seen the article in question."

"Yes," she replied, "it was pointed out to me this morning, and first I should like to say that seven of the male nurses, not four as stated, have gone out again to South Africa."

"Was that a smaller contingent than you wished?"

"Not at all. The committee decided that seven would be sufficient to fulfil the duties required, and these seven men were asked if they cared to return. In each case they were willing to do so."

"How many male nurses sailed on the vessel on her first journey?"

"Eighteen; but the same men would not, in any circumstances, even had the conditions under which the 'Maine' was to work remained unaltered, have all gone out in her. I should not, of course, care to mention names, but it had been decided that two or three of the nurses should not sail on the vessel again, and a few were hardly in sufficiently robust health. One man more especially had quite knocked up."

"Amongst other things, I notice that the male nurses are said to have complained of much discomfort on board."

Lady Randolph smiled as she observed, "It seems to me that living on board ship naturally entails a good deal of discomfort to everyone concerned. I daresay the men may have been asked to do some things which they did not like. But the same thing frequently happens in an ordinary hospital."

"The grievances of the men had not then, I presume, assumed formidable proportions?"

"Decidedly not; and as since their return they appeared before the committee and were given testimonials and a special 'Maine' medal, both of which they all seemed glad enough to receive, it is a little difficult to understand that any serious dissatisfaction prevailed amongst them."

### Death in Our Ranks.

Many of our readers will learn with regret of the death of Miss George, of Dundee East Poorhouse Hospital. Miss George was for some years connected with Mrs. Higginbotham's Nursing Home in Glasgow, leaving there on her appointment as superintendent nurse at the East Poorhouse, which position she held conjointly with that of matron of the hospital since its opening some years ago until her death. Miss George's unselfishness of disposition and brightness of spirit made her much beloved by all with whom she came in contact.

### To Nurses.

We invite contributions from any of our readers, and shall be glad to pay for "Notes on News from the Nursing World," or for articles describing nursing experiences, or dealing with any nursing question from an original point of view. The minimum payment for contributions is 5s., but we welcome interesting contributions of a column, or a page, in length. It may be added that notices of entertainments, presentations, and deaths are not paid for, but, of course, we are always glad to receive them. All rejected manuscripts are returned in due course, and all payments for manuscripts used are made as early as possible at the beginning of each quarter.

### A Picture in Outline.

He was dying in a great London hospital.

Young, gifted, and fair, life had seemed full of promise to him, but mountains of gold had risen between his soul and the vision of the "Great Beyond."

The mountains had crumbled away, for, like the prodigal of old, he had "wasted his substance in riotous living," but his father, unlike the prodigal's, had cast him off, destitute.

And now he was alone—dying!

The doctors had pronounced the case hopeless—"few days at most."

The shadows deepened in the hospital ward, and the heavy rain splashed against the windows, as though God's angels wept to see the sad picture.

And this was the end!

Was it?

He moved, uneasily, and his mind wandered back ten weary years.

He was in a garden amongst the roses, straying down a leafy pathway, hand in hand with the fairest woman he had ever known.

Once again, he looked into her sweet eyes, and watched the sunlight glint on her gold hair, and was happy.

He had been asked, "Are there any relations?" "No."

"Nor friends?" "Yes"—he had hesitated—"there is one."

And the message had been sent from the Free Hospital to the West End mansion.

Would she come? He looked for his watch, which had been pawned, long ago.

"Nurse, what time is it?" "Four o'clock."

He wondered, feverishly, if she would be too late, and closed his eyes.

A stray sunbeam, creeping through the raindrops, roused him, and he looked up, dreamily.

Was he in the garden, again?

A tall, beautiful woman was bending over his bed, her sweet face quivering with love and tenderness.

"Jack!" she whispered, her cool, strong hand clasping his wasted one.

"Margaret!" and love spanned the waste of weary years.

Through a woman's clear eyes he had a glimpse of Paradise, and the hospital ward became the "Gate of Heaven."

### Minor Appointments.

Bolton Infirmary.—Miss Ethel Andrew has been appointed Sister. She was trained at Salford Infirmary, and has since been charge nurse at the Corbett Hospital, out-patient sister and night superintendent at the Stanley Hospital, and ward sister at Northampton Hospital for Women.

Devonport Workhouse.—Miss Elizabeth Nicholls has been appointed Superintendent Nurse. She was trained at Sheffield Union Infirmary, and has since been charge nurse at Park Hospital, Hither Green.

Carlisle Workhouse Infirmary.—Miss Annie Abbott has been appointed Charge Nurse. She was trained at Leeds Union, and has since been nurse at Eccleshall Infirmary, Sheffield.

### Everybody's Opinion.

[Correspondence on all subjects is invited, but we cannot in any way be responsible for the opinions expressed by our correspondents. No communication can be entertained if the name and address of the correspondent is not given, as a guarantee of good faith but not necessarily for publication, or unless one side of the paper only is written on.]

### THE HOURS OF INFIRMARY NURSES.

"A Hard-worked Probationer" writes: As the nurses' off-duty time in some infirmaries is attracting attention, I should like to bring under notice that of Greenwich and St. George's, Fulham, Infirmaries. The nurses work daily from half-past six a.m. till half-past seven p.m., getting four hours off one week, and from two p.m. till ten p.m. alternately, with a pass for church every other Sunday. Comparing this with the off time of other training schools, such as St. Pancras or Lewisham, there is such a vast difference that the question one involuntarily asks, Is the Local Government Board aware of this? Surely not, or they would do justice to all.

### PROMOTION IN INFIRMARIES.

"Interested" writes: I read the letter of "Nurses' Friend," and sympathise deeply with the staff of St. Marylebone Infirmary in the injustice they have been subjected to. No doubt the lady appointed possesses the *sine quâ non* of higher posts in workhouse infirmaries, viz., hospital training. One has only to look round the large infirmaries of the metropolis to see such is the case. I strongly advise educated women who aspire to advancement in the profession to eschew a workhouse infirmary as their school. They will find that one year as paying probationer at one of the large hospitals will carry them to a much higher and more responsible position than ten years of experience in the best workhouse infirmary. Of late years there has been a great deal written and said about the difficulty of finding educated and refined women to take up this work, but the reason can easily be explained if the guardians choose to promote outsiders to the detriment of those in their own schools.

### PRIVATE NURSES AND UNIFORM.

"A Private Nurse" writes: I shall be grateful to you if you will kindly insert this letter in your valuable paper. I am anxious to know whether it is the rule for nurses when joining a co-operation to be restricted to wearing a certain uniform, the colour and style being selected by the superintendent, nothing having been mentioned to the nurses before joining. Being acquainted with several of the nurses on the London co-op., I know that they more or less wear plain clothes, and the only country co-op. that I know of where the principal insists on her nurses dressing to her taste she is disliked because of this arbitrary rule. My experience of two good institutions was this—as uniform was not provided, both allowed the nurses to dress as they wished. It is important to most nurses to save money, so that it is hard, with complete sets of uniform, to be told to procure another simply to please one individual. I am quite certain, if neat and clean and in good uniform, no patient or her friends would express a preference for blue or brown. I should be glad of the general opinion of nurses on this subject. I may add, I am quite aware it is possible to leave the home, but that is not desirable when getting to know the doctors and place, &c.

### THE USE OF THE OBSTETRIC BINDER.

"Monthly Nurse" writes: I should be glad if some maternity nurse of many years' standing would give her experience of the use of the obstetric binder in monthly nursing, as there is a diversity of opinion between doctors and nurses as to its efficacy. When on the staff of a lying-in-hospital I was sent to a "primipara" a fortnight confined, who had secondary hæmorrhage. She had been bound only for twelve hours after delivery, her medical attendant having no faith in binding. Another lady "primipara" had her binder discarded altogether when she first got up at the end of two weeks; while standing up severe hæmorrhage came on, and she had a very long and tedious illness, and has never regained her former strength. In that case the nurse was responsible. I am now nursing a lady "primipara;" the binder was discarded on the twelfth day, and patient being allowed to sit up she took a severe chill, which developed into metritis and pelvic cellulitis. The nurse had previously been nursing for a doctor who did not believe in binding at all. I have been very successful myself keeping the binder on at night for a month, substituting it in the day with a shaped binder with straps round the legs to keep it below the corsets. I feel that if nurses only knew the right thing to do a lot of suffering would be saved. Has a nurse a right to remove the binder without permission of her doctor?

### THE AGE LIMIT.

"Charity" writes: Like "Pavo," I have pleasure in thanking Miss Mary Gardner for her defence of nurses over thirty-five years of age. I agree with her that nothing is more sad in the present state of keen struggle for existence than the thrusting aside in favour of younger competitors those women who have passed their youth, and whose years have doubtless been well spent with God's sick poor. As Miss Gardner says: We cannot be allowed to starve. We must provide against the time when we shall be no longer desired, although "with life's race only half run and the dear human heart still green within us." It seems reasonable that a trustworthy and diligent worker should be allowed to work as long as she is able in the state of life into which it has pleased God to call her. I agree with "E. B.," that a healthy physique and a fine character will scarcely culminate before some two-score years and ten have run, and I feel sure that all readers of Miss Gardner's letter will commend her for her appeal on behalf of old nurses.

### INEXPERIENCED MONTHLY NURSES.

"Nurse Annie" writes: My plan is to place in proper position under the patient a stout mackintosh about four feet square. This I cover with an old sheet folded double, or fourfold if sufficiently large. I prefer old twilled sheets for this purpose. When previously boiled they are aseptic, and after use can have all stains removed by boiling. I find by the use of a receiver that the sheet can be much saved and the bed kept drier and more comfortable to the patient. I use it when the membranes rupture, during the second stage, when there is an excessive flow of liquor amnii, and also when the placenta is being expressed. I prefer the kidney-shaped enamel receiver. I empty it as it becomes filled into a vessel by the side of the bed. After use it can be washed and disinfected. I fail to see the advantage of using absorbent sheets if they require baking to ensure their asepsis. I do not know of any instance in which a surgeon thought it necessary to bake gauze, lint, or wool before using. Are absorbent sheets not prepared similarly and packed with equal care?

### THE ONE THING NEEDFUL.

"Nurse Mary" writes: I thank "Sister" for her reply. In answer, I would not for one moment ignore the thoughtful kindness of Roman Catholic patients praying for the dying around them. I do not doubt but there are many earnest hearts among them, and that such prayers for the well-being of their fellow-sufferers will be answered. Yet, is it right that the spiritual welfare of our Protestants should be so neglected? There is serious fault somewhere. There is also a remedy; the question is, will the remedy be used? Since it is not yet the order that permanent chaplains be appointed to our workhouse infirmaries, I appeal to the Christian nurses of these institutions to faithfully fulfil their own responsibility in this most important matter, and to do all in their power to bring about a better condition of things. I am sure there are many Christian nurses who feel as strongly about this matter as I do, whom, if they were given permission, would most gladly hold a short, bright, evangelical service in their wards morning and evening, remembering that a nurse who has the spiritual, as well as the temporal, needs of her patients at heart has a double privilege.

### For Reading to the Sick.

GOD is our refuge and strength; a very present help in trouble.—*Ps.* xlvi. 1, 2.

Wish not, dear friends, my pain away,
Wish me a wise and thankful heart,
With God in all my griefs to stay,
Nor from His loved correction start.

In life's long sickness evermore
Our thoughts are tossing to and fro;
We change our posture o'er and o'er,
But cannot rest or cheat our woe.

Were it not better to lie still?
Let Him strike home, and bless the rod;
Never so safe as when our will
Yields undiscerned by all but God.

Thy precious things, whate'er they be,
That haunt and vex thee, heart and brain,
Look to the cross, and thou shalt see
How thou mays't turn them all to gain.

The wanderer seeks his native bower,
And we will work and long for Thee,
And thank Thee for each trying hour,
Wishing, not struggling, to be free. —*Keble.*

**Reading.**

We have much leisure for thought in times of helplessness, when we are forced to be idle by weakness of the body: the mind robbed of most of its present interests, and debarred from the cultivation of many intellectual pleasures, turns back upon itself, and goes over page after page of its past history; old memories crowd across our view; old sins, old joys, bygone scenes of our long-past youth.

If our memories are happy, they beguile us of many a weary hour, and wrest from melancholy long stretches of time; and thankful indeed to God may we be that this is so.

If our recollections are sad, especially if saddened by sin, by the memory of many faults, bad habits, evil tempers, time thrown away on light and foolish amusements, talents wasted, selfish plans and deeds, what must we do? They are at times almost unbearable. We reap in saddened memories the harvest sown in days of folly; our past lives look terrible, full of sin, full of mistakes, full of foolishness, even to ourselves. What must they seem to God?

Child of God! for so thou art still if thou art truly penitent; if thou humbly knowest that thy heart, thy will, thy soul, though sometimes failing, are yet striving towards the right. If thou art desirous in the strength of God to persevere and to continue a member of Christ's body . . . then repent! Repent while there is time . . . take thy past unto the Lord again, as thou dost take thy sins, thy cares, thy sorrows, nay, thy very self, and trust Him with it. And He, whose mighty heart is full of love overflowing to His own, He who Himself hath suffered for us, who is "slow to anger" and "of great mercy," He will pardon us, He will never let us go . . . and lead us safely to the bourne of rest where sin itself shall be forgotten and cast out.—*E. A. D.*

I could sit and weep
Over my heart's sorrow,
But on Thine arm Thou bidd'st me sleep
And wait Thy morrow. —*T. Williams.*

### Appointments.

CHESTER INFIRMARY.—Miss Alice G. Cresswell has been appointed Lady Superintendent. She was trained for three years in the Chester Infirmary, and has since been on the private nursing staff two and a-half years; sister, four and a-half years; and senior sister, three years.

### Notes and Queries.

**THE Editor is always willing to answer in this column, without any fee, all reasonable questions, as soon as possible.**
**But the following rules must be carefully observed:—**
1. **Every communication must be accompanied by the name and address of the writer.**
2. **The question must always bear upon nursing, directly or indirectly.**

**If an answer is required by letter a fee of half-a-crown must be enclosed with the note containing the inquiry.**

*Convalescent Homes.*

(53) Will you kindly insert in THE HOSPITAL the address of a convalescent home for nurses in Claremont Park, Blackpool, and also in South Shore, Blackpool? They were advertised in THE HOSPITAL a short time ago.—*Nurse Madeline.*

There is no trace of these homes in the advertisements in THE HOSPITAL since last October.

*Mackintosh.*

(54) I shall be glad if you can inform me how in buying mackintoshes I can tell which are likely to be durable?—*F. C.*

Buy of a reliable firm. The best are usually soft and flexible.

*Cairo.*

(55) Would you kindly inform me what hospitals in or near Cairo employ trained English nurses as sisters, and to whom I should apply for information as to possible vacancies?—*A Nurse.*

The Matron, Kasr-el-Aini Hospital, Cairo, would probably be the best person to whom to apply.

*Friar's Balsam.*

(56) A nurse of some years' standing would be glad of an opinion as to the value of Friar's balsam for bed sores.

Friar's balsam is one of the many mildly antiseptic applications which may be used. Much depends upon the care with which applications are made.

*Probationer.*

(57) I am 21, and wish to become a nurse. I have already had "The Nursing Profession," but the number of applications against the vacancies gives one little hope. I have applied at one hospital for an interview. The answer was that as there was no vacancy it would be useless. Could you advise me? What would be the best course to pursue? I would be an ordinary probationer, and would like a small salary.—*E. M. M.*

A really suitable candidate for training as nurse may have to await a vacancy, but sooner or later she will find one. Your difficulty may be that you are still too young for the institution to which you applied. Write to several hospitals, giving full particulars, and asking when there will be a vacancy. From one or another you will be sure to receive forms of application with full directions how to proceed.

*Hair Cutting.*

(58) Kindly say whether there is any law prohibiting the cutting of children's hair without the consent of parents on admission into a scarlet fever ward?—*Fretus.*

There is no law, and the hospital authorities must necessarily be allowed to exercise their own judgment.

*Dispenser.*

(59) 1. Will the Apothecaries' Hall Certificate qualify for a post of lady dispenser? 2. Where can information be obtained about nursing in the hospital of Umtali, Mashonaland.—*Dispenser.*

1. No; as assistant dispenser only. 2. Write direct to the Matron or Medical Officer at the hospital.

*Salisbury Treatment.*

(60) What is meant by the "Salisbury Treatment"?—*Learner.*

The "Salisbury Treatment" is a system of treating certain disorders by diet.

*Probationer.*

(61) I am most anxious to become a probationer in a children's or general hospital. Would you kindly tell me what books to study? (17.) —*Annie B.*

Matrons as a rule prefer to begin at the beginning in training their probationers. We cannot, therefore, advise you to study nursing subjects from books. But cookery and housewifery in every department is useful to a nurse.

*Paralysis.*

(62) Could you kindly tell me of any home or institution in the neighbourhood of Birmingham where a working man who is suffering from paralysis agitans would be received at a moderate payment, and oblige.—*A. W.*

See reply to District Nurse.

*Guyoscope.*

(63) There is a journal published by Guy's Hospital, London; would you kindly tell me the name, also where it is obtainable?—*M. E.*

*The Guy's Hospital Gazette*, published by F. Ash, 42, Southwark Street, S.E.

**Standard Books of Reference.**

"The Nursing Profession: How and Where to Train." 2s. net.
"The Nurses' Dictionary of Medical Terms." 2s.
"Burdett's Series of Nursing Text-Books." 1s. each.
"A Handbook for Nurses." (Illustrated.) 5s.
"Nursing: Its Theory and Practice." New Edition. 3s. 6d.
"Helps in Sickness and to Health." Fifteenth Thousand. 5s.

All these are published by THE SCIENTIFIC PRESS, Ltd., and may be obtained through any bookseller or direct from the publishers, 28 & 29, Southampton Street, London, W.C.

### Travel Notes.

### XLIX.—IN THE LOIRE COUNTRY.

I am going to describe this part of the world to you with a special view to the requirements of the cyclist, but should you not be votaries of the wheel it will be easy to modify my arrangements to your own needs, and to make the tour with the help of the rail, driving, or on your feet. I cannot tell you a quarter of the charm which lies in the beautiful country of the Loire in my limited space. You can spend six weeks as I did, exploring its charming woods and dells, and revelling in the luxuriant vegetation which clothes the banks of the historic river, and find that you still have left much unseen, but if you have only a fortnight at your disposal much may be done in that time, and the main features of the country studied.

### The Journey and its Cost.

The best way is to go viâ St. Malo and return viâ Paris. First-class ticket to St. Malo £1 15s. 10d.; second class, £1 5s. The latter is very agreeable, and there is positively nothing to object to. From Paris to London, viâ Newhaven and Dieppe, £1 14s. 7d. first class, and £1 5s. 7d. second.

### How to Estimate the Cost of Railway Travelling en Route.

Take a piece of thread and compare the distance you wish to travel with the scale on your guide-book map; it is always placed immediately under the name of the map, and is reckoned in English miles and French kilometres. Every mile is, roughly speaking, a penny, if you travel third class, the same as in England, and for second class you may about double it. This rule, if remembered, you will find very helpful.

The road from St. Malo to Nantes is not profoundly interesting, and you may prefer to take the train; it will occupy you all day, though the distance is not more than a hundred miles. French trains are slow, and there are more changes than on our lines. If you cycle, do it thus: from St. Malo to Rennes forty miles—sleep there—from Rennes to Chateau Briand thirty miles—sleep there—and from Chateau Briand to Nantes, thirty-five miles.

### Starting from Nantes.

Before undertaking this little trip I should advise my travellers to study Theodore Cook's book, called if I remember rightly, "Old Touraine"; it will greatly help to a due appreciation of the wonderful castles and interesting old world towns dotted along the Loire, which still retain so much of the grim simplicity of the past, such as Amboise and Blois, though they, too, have their modern quarters given up to Dame Fashion and the frivolities of the nineteenth century. One night or two will enable you to see the chief objects of interest in Nantes. Foremost I place the massive castle, now used as barracks; with some difficulty, but eventually through the courtesy of the Commandant, I made my way into the interior, and was permitted to sketch in the vast square, and to carry away some reminiscences of the lovely windows, richly canopied, which line the upper half of the walls. Anne of Brittany was married in the chapel to Louis XII., and here, too, Henry IV. in 1598 signed the Edict of Nantes. Some sixty years later Cardinal de Retz was imprisoned in the grim round tower and pluckily made his escape by a rope from an upper window. The cathedral is interesting, though far inferior to many others in France. In the Place is a statue of the gallant Cambronna, author of the famous defiance, "La garde meurt et ne se rends pas!" On the south side of the river, distant some half mile, is still to be seen the ill-omened building "Salorges," now used as a factory, where in 1793 the infamous Carrier murdered nearly 1,000 persons by his republican baptisms and marriages. On leaving Nantes put yourself and machine on board the steamer for Angers, by which means you will see something of the river traffic, which differs considerably from ours.

### Bluebeard's Castle.

You will pass Champtocé, where the engaging ruffian, Gilles de Lavel, lived, the original of the Bluebeard dear to our childish days. He was condemned to be burnt for his crimes in 1440, and suffered in the square at Nantes.

### Angers and Saumur.

Angers is a sombre town. The chateau is a splendid specimen of the feudal fortress; flanked by seventeen towers, it dominates the old town, and seems to throw a sinister shadow on the inhabitants below. Once the capital of Anjou, Angers still retains its walls, its staircased street, impossible to vehicular traffic, and admirably constructed for the purposes of defence; its fifteenth-century timbered houses, and its generally forbidding appearance caused by the black tiles so largely used in its construction. Proceed next day to Saumur, a large cavalry depôt. I think one night will be enough here to study the very remarkable castle and the two ancient churches, but it is a good resting-place from which to visit.

### Chinon, Azay-le-Rideau, and Langeais.

Our Henry II. died at Chinon. Murray calls it the "French Windsor." It is in a ruinous condition, though enough is left to speak of its former splendour. Its position is striking, but all is ruin and decay, and it is a relief to turn to smiling Azay-le-Rideau, where, if your time is not severely limited, it would be pleasant to stay a couple of nights. It is a dream of beauty, and where all are lovely among the Loire chateaux, it stands first in my affections. It is built on the Indre in a wonderfully fertile spot, and on a hot sunny day in June it is entrancing to rest by the roadside and listen to the drowsy hum of the bees and the soft cooing of the wood pigeons. The chateau is of the period of Francis I., and is therefore of the richest and most ornate style of architecture. It is everywhere ornamented with the salamander, Francis' emblem, and the initials of the founder, Gilles Berthelot. The interior, with great good taste, has been left almost unaltered, and shows some fine specimens of ancient furniture, notably a bed, the canopy of which is supported by four carved figures. Six miles distant is Langeais. Here the chateau is less imposing but much older, having been begun in the thirteenth century. The builder, Pierre de Brosse, was hung at Montfaucon for murder and treason, and the castle was completed by Jean Bourré, Minister of Louis XI. Anne of Brittany's marriage to Charles VIII. took place here, by which Brittany was absorbed into the Kingdom of France.

(*To be continued.*)

### TRAVEL NOTES AND QUERIES.

Rules in Regard to Correspondence for this Section.—All questioners must use a pseudonym for publication, but the communication must also bear the writer's own name and address as well, which will be regarded as confidential. All such communications to be addressed "Travel Editor, 'Nursing Mirror,' 28, Southampton Street, Strand." No charge will be made for inserting and answering questions in the inquiry column, and all will be answered in rotation as space permits. If an answer by letter is required, a stamped and addressed envelope must be enclosed, together with 2s. 6d., which fee will be devoted to the objects of the "Hospital Convalescent Fund." Any inquiries reaching the office after Monday cannot be answered in "The Mirror" of the current week.

A Cheap Tour in Wales.—If the lady who wanted to make a short tour in Wales on a small sum will send me a stamped and addressed envelope, I think I have what she wants, but have lost her address.

Paris (Inexperienced).—I should decidedly advise your going with a party. Dr. Lunn's offer great advantages; full particulars were given in The Hospital of April 21st. You would, as an unaccustomed traveller, find much difficulty in getting reasonable accommodation, and would be probably "pushed to the wall."

Scotland (Claymore).—I am afraid that you cannot make a cheap tour in Scotland—at least, not for a short time. If you could remain long enough in each place to take lodgings (if there are any) it might be possible, but not going rapidly from one district to another. I made a trip myself last year on very economical lines, but my hotel expenses were never below 10s. per day. The reason is that food and general necessaries of life in many cases have to be brought long distances, and also that as yet there is not great competition amongst hotels. You may consider yourself clever if you can keep within 10s. per day. I shall be happy to advise you as to routes.

## Figures and Tables

**Fig. 9. f1:**
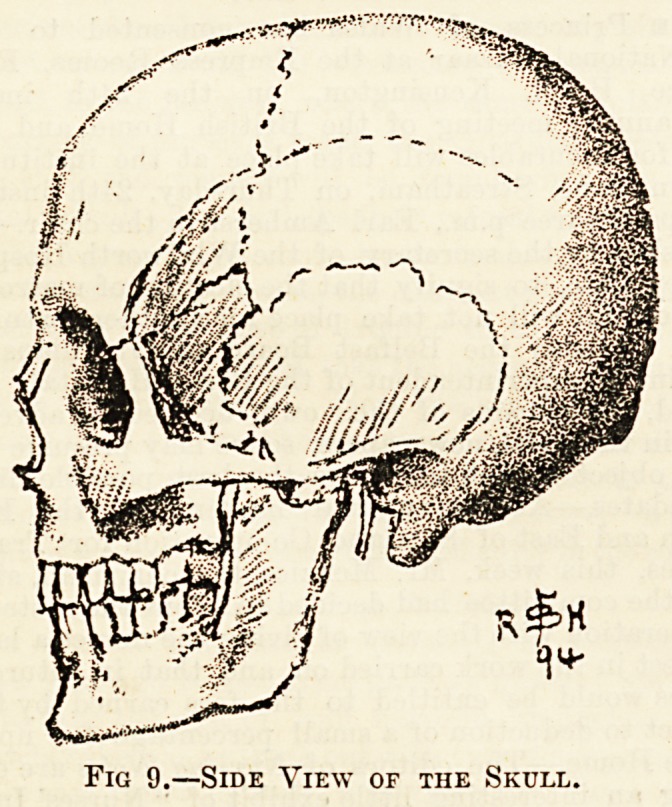


**Fig. 10. f2:**
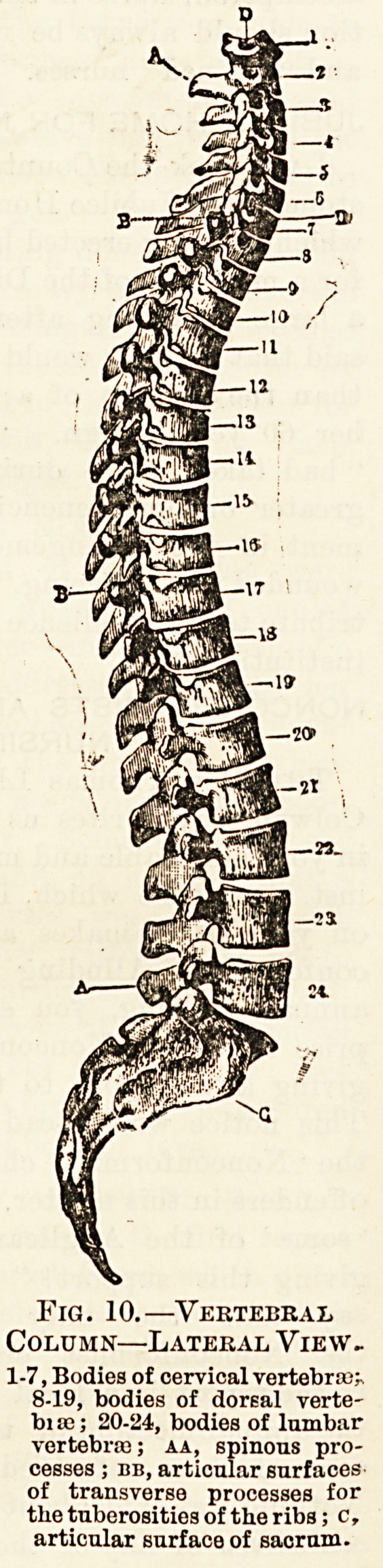


**Fig. 11. f3:**